# Residual Distribution and Risk Assessment of Polychlorinated Biphenyls in Surface Sediments of the Pearl River Delta, South China

**DOI:** 10.1007/s00128-015-1563-z

**Published:** 2015-05-28

**Authors:** Zini Lai, Xiuli Li, Haiyan Li, Lina Zhao, Yanyi Zeng, Chao Wang, Yuan Gao, Qianfu Liu

**Affiliations:** Pearl River Fisheries Research Institute, Chinese Academy of Fishery Sciences, Fishery Ecoenvironment Monitor and Evaluation Function Laboratory of Pearl River Valley, Guangzhou, 510380 China; Shanghai Ocean University, College of Aquatic and Life, Shanghai, 201306 China

**Keywords:** Pearl River Delta, PCBs, Surface sediments, Risk assessment

## Abstract

We analyzed residual PCBs in surface sediments at 19 sites in the Pearl River Delta in the wet and dry seasons. Seven indicative PCB congeners (PCB28, PCB52, PCB101, PCB118, PCB153, PCB138 and PCB180) were detected in the surface sediments, among which the detection rate and mass concentrations of PCB52 were the highest. Total concentrations of the seven PCBs ranged from 19.8 to 111 μg/kg, with an average of 48.2 μg/kg. For the spatial distribution, the sum of the seven PCB (∑PCB) concentrations for the stations that were located in the city region of the Pearl River Delta were significantly higher than the ∑PCB concentrations for the eight outlets of the Pearl River Delta (*p* < 0.05). According to the US National Oceanic and Atmospheric Administration ERL and ERM guideline concentrations, the PCB concentrations may occasionally lead to adverse effects, especially in the dry season.

Polychlorinated biphenyls (PCBs) are a group of 209 different chemicals considered to be pollutants of environmental and human health concern. Although PCBs are now ubiquitous in the environment, they have resulted from industrial production without any known natural source. Atmospheric transport and deposition, current transport, riverine input, sea-ice transport and biotic transport are considered to be the main sources (Macdonald et al. 2000; AMAP 2004). PCBs were widely used in many manufacturing processes between the 1930s and 1970s, and their production rose quickly, reaching 2.1 × 10^6^ t to satisfy worldwide demand. It is reported that approximately 10000t of PCBs were produced from 1965 to the early 1980s in China, accounting for about 1 % of global production (Zheng et al. 2010). Although the production of PCBs has been banned since the early 1970s in many countries, residuals can still be found in the environment (Harrad et al. 1994; Sprovieri et al. 2007). PCBs persist as legacy pollutants for which chronic toxicity still represents a serious environmental risk due to their stability and permanence (Wang et al. 2011; Konat et al. 2001).

The Pearl River Delta (PRD) is located on the southeast coast of China. It is one of the most developed regions and also the second most populous area in China, resulting in environmental pollution that may affect the health of the population in the region. Electronics, electrical machines, and petrochemicals dominate the local industrial structure, cumulatively accounting for over 50 % of the local industry. The urbanized and industrialized processes in the region, especially from electrical factory industrial activities were assumed to be the main sources of PCBs (Kang et al. 2000; Mai et al. 2005; Nie et al. 2005, 2006). PCB contamination in the region was mainly concentrated in the sediment and water (Yang et al. 1997; Kang et al. 2000; Mai et al. 2005;). Numberous studies have investigated the occurrence of PCBs in various compartments of the PRD (Nie et al. 2005; Wang et al. 2011; Zhang et al. 2013; Guan et al. 2009).

The Global Environment Monitoring System – Food Contamination Monitoring and Assessment Programme (GEMS/Food) stipulate PCB28, PCB52, PCB101, PCB118, PCB138, PCB153 and PCB180 as indicator PCBs to indicate PCBs pollution status. Zhang et al. (2013) reported that six of these PCBs (PCB28, 52, 101, 118, 138, and 153) were detected in most surface soil samples in the PRD. The present study covers the whole Pearl River watershed (19 sites), while other papers focus on just one part of the watershed. The distribution of the seven indicator PCBs was determined in recent sediments from the PRD in this study. The present study aimed to assess the current contamination level and evaluate the temporal variation that occurred over the decades. The composition and distribution of the PCBs was investigated, and a pollution risk assessment was undertaken. Basic information for the potential hazard assessment of PCBs in the PRD coastal environment was obtained during this study.

## Materials and Methods

**Fig. 1:**
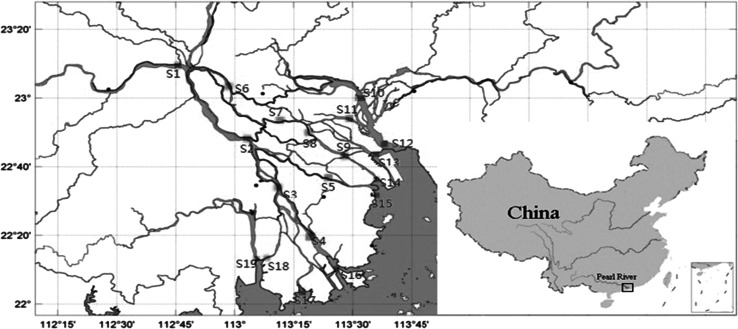
Distribution of the sampling sites in the Pearl River Delta

Thirty-eight samples of surface sediments from 19 sites (Fig. 1) were collected (March 19th, 2012 and August 19th, 2012) from the PRD in south China. The two sampling periods were defined as the wet and dry seasons, respectively. Eleven of the 19 sampling sites (S1–S11) were located in the city region of the PRD, with the other eight sites (S12–S19) being in the outlets of the PRD. The surface sediments were collected with a grab-type sampler, placed into pre-cleaned amber bottles, and stored at –20°C prior to analysis.

**Fig. 2:**
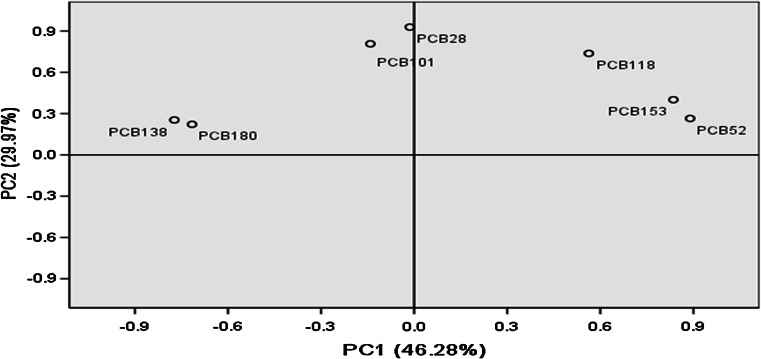
Principal component plot of PCBs in the Pearl River Delta

All the sediment samples from the same site were mixed, freeze-dried, and homogenized by grinding. Firstly, eachmixed sample (5 g) was treated with 40 mL of hexane–acetone (1:1, v/v) for 12 h in a 100 mL colorimetric tube with a stopper. Next, all samples were sonicated for 20 min. Then, the extracts were decanted, and the remainder resonicated with 20 mL of hexane for 20 min (repeated three times). After resonicating, the four extracts were mixed together and treated with activated copper, to remove sulfates. The mixture was then concentrated to 5.0 mL by a rotary evaporator. Next, a separatory funnel was used to remove the extract. Concentrated sulfuric acid (30 mL, 98 %, AR) was added to remove impurities; this was repeated if the extract remained coloured. Ultrapure water (100 mL) was then used to wash the organic phase twice, and the washed organic phase was purified with 5 g of anhydrous sodium sulfate (heated at 550°C for 4 h prior to use). Finally, the sample was concentrated to 1 mL for analysis (Fig. 2).

PCB congeners were analyzed by electron capture gas chromatography (Aglient 6890, Aglient Technologies, Santa Clara, CA, USA). Separations were carried out using an HP-5 quartz capillary column (30 m × 0.32 mm × 0.25 µm). Nitrogen (99.99 % pure) was used as the carrier gas at a constant flow of 1 mL/min. The oven temperature was initially 150°C, held for 1 min, and was increased to 200°C at a rate of 10°C/min, maintained for 1.0 min, and then raised to 260°C at a rate of 5°C/min, and held for 1 min. The injection was at 250°C in splitless mode, and the detector was at 300°C.

A total organic carbon (TOC) analysis was conducted using the National Environmental Protection Standard’s spectrophotometric method (HJ615-2011) for soil-determination of organic carbon-potassium dichromate oxidation.

Strict quality control procedures were implemented throughout the entire process. For every set of 10 samples, procedural blanks, spiked blanks (standards spiked into solvent), matrix spikes/matrix spike duplicates, and sample duplicates were processed. The spiked recovery for PCB congeners in the sediments ranged from 80.9 % to 113.2 %. The instrumental limit of detection (LOD) was determined as the concentration of analytes that gave rise to a peak with a signal-to-noise ratio (S/N) of 3, and ranged from 0.01 to 0.06 μg/kg for the different PCB congeners. All the data were corrected with the recovery rates and reported in μg/kg dw (dry weight).

The ∑PCB concentration was defined as the sum of seven PCB congeners (PCB28, PCB52, PCB101, PCB118, PCB138, PCB153 and PCB180). Data analyses were performed using SPSS 14.0 (SPSS Inc., Chicago, 1L, USA). Multiple comparisons were conducted with Tukey’s honestly significant difference (HSD) test using ANOVA. Differences were considered to be significant if *p* < 0.05.

## Results and Discussion

As shown in Table 1, the seven indicative PCB congeners had different detection rates in the sediment samples from the PRD in the wet and dry periods. The ∑PCB concentrations in the sediments ranged from 19.8 to 111 μg/kg, with an average of 48.3 μg/kg. The detection rate and concentrations of PCB52 were the highest: the detection rate of PCB52 was 100 %, followed by PCB180, with a detection rate of 97.4 %; the concentration of each PCB congener was in the order PCB52 > PCB118 > PCB101 > PCB153 > PCB28 > PCB138 > PCB180.

**Table 1:** Concentrations of PCBs in surface sediments from the Pearl River Delta

PCB	Number of chlorine atoms	Range (μg/kg)	Average (μg/kg)	Detection rate (%)
PCB28	3	ND–14.5	2.65	52.6
PCB52	4	9.90–53.2	27.2	100
PCB101	5	ND–13.2	3.54	65.8
PCB118	5	ND–32.7	7.05	81.6
PCB138	6	ND–15.1	2.46	71.1
PCB153	6	ND–15.0	3.33	89.5
PCB180	7	ND–11.4	1.96	97.4

*ND* not detected

Studies have shown that incinerators and processes using chloride oxidation (e.g. paper bleaching or de-inking technology) discharge predominantly less-chlorinated PCBs (Krauss and Wilcke 2001); however, the pollutants in transformer oil have more highly chlorinated PCBs (Takasuga et al. 2006). The concentrations of less-chlorinated PCBs were higher than those of highly chlorinated PCBs in the sediment samples from the PRD in the two periods. The tetra-PCB content accounted for the largest proportion of the total amount of sediment PCBs, at 56 %. This implies that the PCBs in the PRD may be related to the wastewater from nearby paper mills and dyeing and weaving works; and perhaps also because highly chlorinated PCBs could be decomposed to less-chlorinated PCBs by bacteria, fungi and other microorganisms. However, an additional explanation may be that the major PCB congeners discharged into the environment in China were tri-PCBs to hexa-PCBs and comprised 80 % of the total PCB production. Furthermore, it is easier for less-chlorinated congeners to be transported over long distances (Wu et al. 2011).

The distribution of PCBs in the PRD sediments, with concentrations of highly chlorinated PCBs relatively low in the study area, is in accord with many other studies (Shen et al. 2006; Chen et al. 2009; Ji et al. 2009). Guan et al. (2009) studied PCBs in riverine runoff of the PRD, and found that tri- to penta-PCBs accounted for approximately 90 % of total PCBs. No octa- to deca-PCBs were detected. Chen and colleagues (1999) analyzed sediment samples for 14 PCB congeners from 22 rivers in east China, and found PCB52, PCB101, PCB87 and PCB149 (i.e., tetra-, penta- and hexa-PCBs) to have the highest concentrations, accounting for 50 % of the PCBs. Zhang et al. (2013) analyzed PCB contamination in soils of the PRD, finding that PCBs were dominated by low-chlorinated biphenyls; however, the pro-portion of higher-chlorinated biphenyls was elevated with the influence of industrial activities.

Concentrations of ∑PCBs and TOC in August in surface sediments from the PRD are shown in Table 2. A correlation analysis between the ∑PCBs concentrations and TOC at each site revealed significant correlation (r = 0.550, *p* < 0.05). The findings were consistent with a previous study which indicated that higher concentrations of PCBs typically occur in sediments having a larger fraction of clays, OM, or micro-particulate matter (Burgess et al. 2001), but not consistent with another study which found that ∑PCBs concentrations did not significantly correlated with their geochemical parameters (including sediment carbon) (Wang et al. 2011).

**Table 2:** Concentrations of ∑PCBs and TOC in August in surface sediments from the Pearl River Delta

Stations	∑PCBs (μg/kg)	TOC (%)
S1	45	1.96
S2	35.2	2.28
S3	44.6	2.03
S4	37.3	2.32
S5	48.5	1.34
S6	67.6	1.34
S7	48.2	1.81
S8	85.5	2.04
S9	47	1.49
S10	94.1	1.24
S11	48.1	1.60
S12	42.9	2.16
S13	30.7	2.14
S14	34.7	2.56
S15	29.5	2.76
S16	35.7	2.00
S17	23.9	2.00
S18	29.9	1.68
S19	37.4	1.98

The first two principal components (PCs) were extracted by PCA, relating to 46.3 % and 30.0 % of the total variance, respectively. PCB52, PCB118 and PCB153 were grouped together, indicating that they had a similar nature, and might have the same source. In addition, PCB28 was near PCB101, indicating that both PCBs might originate from similar sources; the same applies to PCB138 and PCB180. Analysis showed (n = 19) no correlation between the seven indicative PCBs and the organic matter in sediments. No apparent co-relationships between PCB concentrations and sediment properties were obtained, indicating that the distribution of PCBs was controlled not only by their source but also by multiple factors such as atmospheric transport and deposition, mixing, partitioning and sorption in the water column and sediments (Hong et al. 2012).

**Fig. 3:**
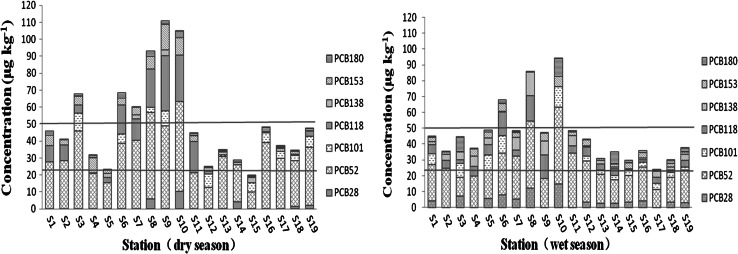
Concentrations of the individual PCB homologues in the wet and dry seasons. (The two *lines* represnt the ERL and the concentration of 50 μg/kg dw, respectively)

The lowest ∑PCB concentrations were detected at site S17 in both the wet and dry seasons; and, combined for both seasons, the highest ∑PCB concentrations were for site S10 (Fig. 3). The ∑PCB concentrations for the sites located in the city region of the PRD (S1–S11) were significantly higher than the ∑PCB concentrations for the eight outlets of the PRD (S12–S19) (*p* < 0.05). However, there were no significant differences between the ∑PCB concentrations for the eight outlets of the PRD (S12–S19) (*p* > 0.05). The highest ∑PCB concentrations were for site S10, mainly because most of the domestic and industrial wastewater from the cities of Guangzhou and Dongguan enter the South China Sea at site S10. According to official statistics, there were more than 230 sewage treatment plants in Guangdong province at the end of 2009, disposing of approximately 13 × 10^6^ t sewage every day. The sewage from the nine cities in the PRD accounted for about 90 % of the total wastewater of Guangdong province. PCBs in water can sink downward into the sediments after a time; therefore, the sewage treatment plants might be the cause of the higher concentrations of PCBs in the city region of the PRD (S1–S11). Additionally, during the operation of the port and shipping, the emission of toxic liquid waste, solid rubbish and fuel oil increased the pollution load of PCBs in the sediments. The ∑PCB concentrations for the eight outlets of the PRD (S12–S19) were relatively low, which may be because the eight outlets accepted not only a huge pollution from the Pearl River stream but also a large quantity of water from the South China Sea that was less contaminated, so that the ∑PCB concentration could be diluted in the water.

It has been reported that PCBs in the soil at the scrap capacitor sealing point in Yangjiang city in Guangdong province were mainly tetra-PCBs, and that their composition was similar to Aroclor 1248 (Chen et al. 2008). A similar composition in the Pearl River reach sediment column has also been observed (Mai et al. 2005), suggesting that imported capacitors may account for a certain proportion of capacitive devices in the PRD, and that the release of PCBs from these components influenced PCB composition in the PRD. The cluster analysis for samples from the 19 sites in this study showed that they had similar compositions, indicating that they may have the same sources, and may be the result of pollution by the same products. However, the influence of many changes that have occurred in the environment cannot be excluded.

The ∑PCB concentrations in the sediments ranged from 19.8 to 111 μg/kg, with an average of 48.2 μg/kg. Compared to other areas around the world (Table 3), the ∑PCB concentrations in the PRD were in the mid-range (Yang et al. 2009; Wu et al. 2011).

**Table 3:** Concentrations of PCBs in surface sediment samples and other reported studies

Location	n	Range (μg/kg dw)	Average (μg/kg dw)	Year	References
Pearl River Estuary, China	128	11.5–485	–	1997	Kang et al. (2000)
Hong Kong coast		0.1–461	52.2	–	Wong et al. (2000)
Pearl River Estuary, China	25	10–303		1997	Mai et al. (2002)
Daya Bay	12	0.85–27.4	8.83	1999	Zhou et al. (2001)
New Brunswick, Canada	132	1.07–10.4	–	1988	Sather et al. (2006)
Pearl River Estuary, China	37	5.10–11.0	7.96	2009	Wang et al. (2011)
Pearl River Estuary, China	36	11.1–23.2		2000	Nie et al. (2005)
Fu River, China	12	4.2–198	46.3	2008	Hu et al. (2010)
Fenhe Reservoir and Watershed, China	123	ND–126.5	27.3	2010	Li et al. (2012)
Haihe River, China	32	ND–253	66.8	2007	Zhao et al. (2010)
Dianchi Lake, China	6	0.6–2.4	1.2	2008	Wan et al. (2011)
The mid- and downstream of the Yellow River, north China	–	ND–6.0	3.1	2004	He et al. (2006)
Wuhan reach of the Yangtze River, China	39	1.2–45.1	9.2	2005	Yang et al. (2009)
Donggang River, Taiwan	121	25.5–63.5	–	2003–2004	Hsieh et al. (2011)
Lake Michigan, USA	163	53–35,000	7400	2006	Martinez et al. (2010)
Bering Sea	14	22–150	71	–	Wang et al. (2012)
Chukchi Sea	14	60–640	190	2008	Hong et al. (2012)
Canada Basin	14	24–600	150	–	Wang et al. (2012)
Pearl River Delta, China	7	19.8–111	48.2	2012	This study

*ND* not detected

As Fig. 3 shows, the ∑PCB concentrations in the wet season were higher than in the dry season, but not significantly (*p* > 0.05). Precipitation and pollution sources are considered to be the main factors causing fluctuations in water quality. The temporal variations in the PCBs in the two periods might be caused mainly by the difference in the amount of precipitation in these two periods (Li et al. 2012). In the wet season, the grain size composition of sediments changed from clay to sand owing to the floods and heavy rains. As a result, the organic matter content in sediments was reduced, so that the content of PCBs in the sediments also decreased. However, in the dry season, with less precipitation and surface runoff, PCBs in the water were deposited in the sediments, and the PCB content of the sediments thus increased.

Considering the toxicity and bioaccumulation property of PCBs, as well as the Pearl River being a major source of water for irrigation and aquaculture, it is of particular interest to evaluate the potential risk of PCBs in sediments of the Pearl River. So far, there is still no uniform standard available to assess the biological effects of PCBs, but several studies have been carried out, and some useful indicators have been provided. Hakanson (1980) established a potential ecological risk index in which the concentration of PCBs is one of the main parameters. The US Environmental Protection Agency and the National Oceanic and Atmospheric Administration (NOAA) have established threshold (TEC) and extreme (EEC) effect concentration sediment quality guidelines (SQGs) for marine sediments (Long et al. 1995; Gomez-Gutierrez et al. 2007). The NOAA guidelines specify the ‘effects range low’ (ERL) and the ‘effects range median’ (ERM), with the ERL (22.7 μg/kg dw) representing the chemical concentration below which adverse effects would rarely be observed, and the ERM (180 μg/kg dw) representing the concentration above which adverse effects would frequently occur. According to Long et al. (1995), the ERL and ERM relate to seven PCB congeners (PCB28, PCB52, PCB101, PCB118, PCB138, PCB153 and PCB180), and ∑PCB concentrations of more than 50 μg/kg dw indicate moderate to severe pollution (Guo et al. 2011).

For PCBs in sediments of the PRD, the average ∑PCB concentration was higher than the ERL but lower than the ERM in both the wet and dry seasons. This suggests that PCBs might cause adverse biological effects on occasion. However, the ∑PCB concentration was more than 50 μg/kg dw in the dry season, indicating that the PRD suffered from moderate to severe pollution in the dry season. Regarding the ∑PCB concentration at each site in the two periods, comparisons of the ∑PCB concentration with the ERL (22.7 μg/kg dw) can be found in Fig. 3. The ERM (180 μg/kg dw) is not shown in Fig. 3. All 19 sites had higher concentrations than the ERL but lower than the ERM in both the wet and dry seasons, except for site S15, which had a lower concentration than the ERL in the dry season. Of the total of 19 sites, 15.8 % had a ∑PCB concentration greater than 50 μg/kg in the wet season, and 31.6 % had ∑PCB concentrations greater than 50 μg/kg in the dry season. These findings could indicate that PCBs in the sediments of the PRD might lead to occasional adverse effects, especially in the dry season.

We evaluated the concentration, composition and spatial distributions of PCBs in the surface sediments of the PRD. In conclusion, the ∑PCB concentrations in the sediments of the investigated area were in the mid-range compared with other areas of China and other countries. In terms of the distribution of different chlorinated PCBs, tetra-PCBs congener (PCB52) was dominant. Regarding the spatial distribution, ∑PCB concentrations for sites located in the city region of the PRD were significantly higher than concentrations for the eight outlets of the PRD (*p* < 0.05). Considering temporal variation, ∑PCB concentrations in the dry season were higher than those in the wet season. In addition, no apparent co-relationships between concentrations of individual PCB congeners and sediment properties were obtained. However, a significant correlation was obtained for the ∑PCB abd TOC during the dry season. Comparison of the measured PCB concentrations to ERL and ERM guidelines indicated that PCBs in the sediments of the investigated area may occasionally lead to adverse effects, especially in the dry season. Therefore, it is important to control PCB contamination in sediments in the PRD.
